# Nitrogen fertilizer improves *Salix matsudana* growth and soil qualities

**DOI:** 10.3389/fmicb.2025.1631852

**Published:** 2025-08-12

**Authors:** Qian Wang, Xiaoyun Niu, Shuo Huang, Dongliu Di, Beibei Su, Yangchen Yuan, Yumeng Wu, Dazhuang Huang

**Affiliations:** ^1^Hebei Key Laboratory of Floral Biological Breeding, Hebei Agricultural University, Baoding, China; ^2^Research Institute of Subtropical Forestry, Chinese Academy of Forestry, Hangzhou, China; ^3^Hongyashan State-Owned Forest Farm, Baoding, China

**Keywords:** *Salix matsudana*, phytoremediation, nitrogen fertilization, rhizosphere microorganism, metabolomic

## Abstract

**Introduction:**

Soil contamination with heavy metals (e.g., Pb, Cd) poses severe environmental risks due to industrialization. *Salix matsudana*, a metal-tolerant woody plant, shows promise for phytoremediation, yet the synergistic role of nitrogen (N) fertilization in enhancing plant growth and soil remediation remains unclear. This study aims to elucidate how N fertilization optimizes *S. matsudana’s* remediation efficiency.

**Methods:**

We applied integrated physiological and multi-omics approaches to assess N fertilization effects on *S. matsudana* growth, Pb/Cd uptake, and rhizosphere properties. Physiological metrics (biomass, metal accumulation) were combined with microbial community analysis (16S rRNA sequencing) and metabolomic profiling (LC-MS/GC-MS) of rhizosphere soils under varying N concentrations.

**Results:**

High N levels significantly increased plant biomass and Pb/Cd accumulation. Microbial diversity shifted, with enriched metal-mobilizing taxa. Metabolomics revealed elevated organic acids, correlating with improved metal bioavailability and soil health.

**Discussion:**

N fertilization synergistically enhances phytoremediation by: (1) stimulating plant growth and metal uptake, (2) reshaping rhizosphere microbiomes for metal mobilization, and (3) promoting chelating metabolite secretion. These findings provide actionable insights for optimizing N-assisted phytoremediation strategies.

## Introduction

*Salix matsudana* (Chinese willow) has emerged as a promising woody energy crop, widely recognized for its applications in landscaping and phytoremediation due to its notable tolerance to heavy metals. This species possesses robust physiological mechanisms that enable it to mitigate metal toxicity, making it a highly suitable candidate for environmental restoration. For example, studies have shown that calcium (Ca) can alleviate cadmium (Cd)-induced toxicity in *S. matsudana* by regulating Cd mobility and enhancing detoxification processes (Zou et al., 2023; Xu et al., 2025). The genetic characteristics associated with *S. matsudana’*s growth and metal tolerance have been extensively studied, highlighting its potential for coastal afforestation and the remediation of heavy metal-contaminated soils (Zhang et al., 2017; Chen et al., 2022). Importantly, its high biomass production and efficient accumulation of metals render it particularly valuable for phytoremediation strategies (Hauptvogl et al., 2020; Liang et al., 2024). Moreover, research has identified key anatomical and physiological adaptations in response to heavy metal exposure, such as modifications in root morphology and elemental distribution, which significantly influence metal uptake and tolerance (Liu et al., 2023; Vaculík et al., 2012). Given these traits, *S. matsudana* serves as a dual-purpose species, offering significant benefits in both bioenergy production and environmental remediation. Its rapid growth rate, substantial biomass yield, and exceptional tolerance to heavy metals establish it as a critical resource for sustainable land management and the rehabilitation of contaminated soils.

The accumulation of heavy metals, particularly lead (Pb) and cadmium (Cd), in agricultural soils has become a critical environmental concern due to the rapid industrialization and urbanization (Zhou et al., 2024; Aziz et al., 2023; Chen Z. et al., 2024; Chen J. et al., 2024). A substantial body of research has explored the sources of contamination, ecological consequences, and potential remediation strategies for these toxic elements (Karthikayini et al., 2024; Kumari and Chakraborty, 2024; Saleem et al., 2024; Yao et al., 2024; Kravchenko et al., 2025; Proshad et al., 2025). Studies indicate that the region’s diverse physiographic and climatic conditions have led to the formation of various soil types, including Entisols, Inceptisols, Mollisols, and Alfisols. However, unsustainable soil management practices have significantly diminished soil fertility, causing widespread deficiencies in essential nutrients while concurrently intensifying the accumulation of hazardous heavy metals such as Cd and Pb. These metals not only impair soil fertility by disrupting key nutrient cycles but also accumulate abnormally within soil systems (Anup et al., 2015). The resultant metal toxicity presents serious risks to both ecosystem health and agricultural productivity. The growing global population and intensified industrial activities have accelerated the buildup of toxic heavy metals, posing severe threats to environmental integrity and public health. This situation highlights the urgent need for effective remediation approaches, among which bioremediation techniques have shown considerable promise by utilizing biological mechanisms to alleviate heavy metal stress (Anup et al., 2015). Notably, heavy metals such as arsenic (As), cadmium (Cd), chromium (Cr), lead (Pb), and mercury (Hg) are widely recognized for their high toxicity and are classified as priority pollutants due to their significant implications for public health (Aziz et al., 2023). Compounding the issue, non-essential metals like Cd and Pb have no known physiological functions, which makes their persistence in agricultural and urban soils particularly challenging to manage. The impacts of heavy metal pollution extend beyond soil degradation, significantly disrupting ecosystems and endangering human well-being. Numerous studies have identified industrial activities and vehicular emissions as primary sources of heavy metal contamination in urban environments, resulting the accumulation of toxic elements such as Pb and Cd in soils. Given the considerable spatial variability in contamination levels—driven by localized environmental conditions and anthropogenic influences—the implementation of comprehensive monitoring and mitigation strategies is essential. The increasing accumulation of heavy metals in soils poses a major environmental and public health challenge, which is further intensified by rapid industrialization and urban expansion. Addressing this issue necessitates a multidimensional strategy that integrates advanced monitoring systems, targeted remediation measures, and robust public health protections. Moreover, continued research into bioremediation and other innovative technologies will be crucial in developing sustainable solutions to combat soil contamination, ensuring long-term ecosystem resilience and human safety.

Nitrogen fertilizers play a crucial role in enhancing plant growth by increasing nitrogen availability, which is an essential macronutrient for plant development (Zhao et al., 2025; Ge et al., 2025; Jatana et al., 2025; Oguz and Arslan, 2025; Sakuma et al., 2025; Song et al., 2025). For instance, studies have demonstrated that the combined application of nitrogen fertilizers and rice straw biochar can significantly improve both the growth and yield of rice (Huang et al., 2017). In addition to increasing crop productivity, biochar amendments have been shown to mitigate nitrogen toxicity while simultaneously improving soil fertility and nitrogen use efficiency (NUE), thereby further supporting sustainable agricultural practices (Khan et al., 2021). Furthermore, the interaction between nitrogen fertilizers and soil microbial communities represents a key factor influencing plant growth dynamics. Research has indicated that specific microbial phylotypes exhibit differential responses to various fertilizer regimes, with their metabolic activities directly affecting soil quality and plant health (Yan et al., 2020). Notably, beneficial microbial genera such as *Sphingomonas* and *Pseudomonas* may enhance the *S. matsudana* growth under nitrogen fertilization by facilitating nutrient uptake and promoting overall plant vigor.

*S. matsudana*, a woody energy tree commonly utilized in landscaping, has been demonstrated to benefit from nitrogen fertilization through enhanced growth. However, the mechanisms by which nitrogen fertilizers promote the *S. matsudana* growth and soil remediation remain insufficiently elucidated. To address this knowledge gap, we examined the effects of varying nitrogen fertilizer concentrations on the *S. matsudana* growth, soil physiological properties, rhizosphere microbial communities, and soil metabolomic profiles. Our results revealed that high nitrogen levels significantly promoted the *S. matsudana* growth and increased its uptake of Pb and Cd. Further root physiological analysis indicated that nitrogen application enhanced Pb and Cd absorption compared to the control treatment. In addition, rhizosphere microbial analysis showed that nitrogen fertilization stimulated microbial activity, thereby enhancing uptake of heavy metals. Metabolomic profiling further confirmed improvements in soil quality under high nitrogen conditions, aligning with the observed physiological responses. These findings offer new insights into the mechanisms through which nitrogen fertilization enhances both plant growth and soil remediation in *S. matsudana*, suggesting potential applications in sustainable landscaping and phytoremediation strategies.

## Materials and methods

### Experimental design

In this study, the effects of nitrogen fertilization on the *S. matsudana* growth and uptake of heavy metals were examined using five nitrogen concentration levels: control (no nitrogen), low, medium, high, and hyper-high. The control (CK) group represents roots grown in heavy metal-contaminated soil without nitrogen fertilizer application. These treatments were applied in four different nitrogen forms—ammonium nitrogen (AM), nitrate nitrogen (NI), a 1:1 combination of ammonium and nitrate (AN), and urea nitrogen (CN) (Cheng et al., 2020; Chen K. X. et al., 2024). Two-year-old *S. matsudana* seedlings were used as the experimental material. The nitrogen application rates were determined based on the standard annual dosage of 120 kg·ha^−1^ (Table 1). All chemical reagents utilized in the experiment were of analytical grade.

**Table 1:** Fertilization treatments.

Treatments	Nitrogen levels (kg mg^−2^ year^−1^)				
	0	60	120	200	300
Ammonium nitrogen	CK	LAM	MAM	HAM	HHAM
Nitrate-nitrogen		LNI	MNI	HNI	HHNI
Ammonium and nitrate 1:1		LAN	MAN	HAN	HHAN
CO(NH_2_)_2_		LCN	MCN	HCN	HHCN

As shown in Table 1, five nitrogen fertilizer levels were established to systematically evaluate the effects of varying nitrogen application rates on the *S. matsudana* growth and uptake of heavy metals. Each treatment was replicated four times to ensure statistical validity. Two-year-old *S. matsudana* saplings, with an average stem diameter of approximately 2 cm, were uniformly pruned to a height of 40 cm and air-dried prior to transplantation. To minimize the influence of residual soil, the saplings were transplanted bare-rooted into pots containing test soil spiked with Pb and Cd.

For the cultivation of potted *S. matsudana*, uniform growth conditions were maintained by using identical pots, each containing 25 kg of soil. The experiment consisted of 17 treatments, including a control group, with one pot per treatment allocated to each of the 17 experimental groups. A randomized block design was employed, and the pots were repositioned biweekly in a randomized manner to minimize positional bias and ensure the comparability of treatment effects across all groups.

Nitrogen fertilizer was applied quarterly in equal portions at 10-day intervals. The fertilizer solution was evenly sprayed onto the soil surface of each pot using a handheld sprayer, immediately followed by irrigation. To ensure consistent growing conditions throughout the experimental period, all pots were randomly arranged in a well-ventilated greenhouse and subjected to uniform management practices, including watering, soil aeration, weed control, and pest management. After 210 days of growth, a pruning procedure was conducted, during which each *S. matsudana* plant was retained with only four vigorous branches, trimmed to approximately 25 cm in length. All removed plant material was collected and disposed of appropriately. Standardized winterization procedures were implemented for overwintering, and nitrogen application treatments were continued into the second year of the experiment. To prevent leaching of Cd and Pb, each pot was fitted with a plastic collection tray. Any leachate generated from irrigation was carefully collected and returned to its corresponding pot to maintain elemental balance. The total cultivation period prior to final harvest was 570 days. The work flowchart was shown in Supplementary Figure S1.

### Plant materials and growth conditions

Two-year-old *S. matsudana* branches of uniform size were collected from the nursery at the Experimental Station of Hebei Agricultural University (38°45′21″N, 115°24′37″E), located in Qingyuan District, Baoding. The region has an average annual temperature of 13°C. In June 2020, branches measuring 40 cm in length were planted in pots (34-cm diameter × 25-cm height) containing 20 kg of meadow brown soil (topsoil, 0–20-cm depth).

### Soil preparation and experimental conditions

Soil properties and metal concentrations before planting were shown in Supplementary Table S1. The soil had been pre-treated with CdCl₂ and Pb(NO₃)₂ more than 5 years prior to the commencement of the experiment, resulting in residual Cd and Pb concentrations of 8 mg kg^−1^ and 220 mg kg^−1^, respectively (Lu et al., 2016; Peng et al., 2022; Liu et al., 2022). During the entire experimental period, soil moisture was maintained at approximately 60% of the maximum field capacity.

### Plant physiology analysis

The entire *S. matsudana* plant was harvested following nitrogen fertilizer treatment and subjected to tissue separation. The collected plant materials—including leaves, branches, stems, stem plugs, and roots—were initially rinsed with tap water and subsequently immersed in a 20 mmol/L Na₂EDTA solution for 15 min to remove surface-adsorbed heavy metal ions, particularly from the root tissues. Following this, the samples were thoroughly washed multiple times with ultrapure water, and excess surface moisture was carefully removed using filter paper. For biomass determination, fresh samples were first oven-dried at 90°C for 30 min, followed by continued drying at 65°C until a constant dry weight was achieved. After weighing and recording the biomass, the dried samples were cut into smaller pieces, ground into fine powder, and sieved through a 60-mesh nylon sieve. The resulting material was then sealed and stored under appropriate conditions for subsequent analysis.

Following pretreatment, approximately 1 g of plant tissue powder was accurately weighed and digested using a HNO₃/HClO₄ acid mixture (4:1, v/v) at 170°C for 3.5 h, until a clear and transparent solution was obtained. The resulting digested solution was filtered through a 0.45-μm membrane filter, diluted to the appropriate concentration, and adjusted to the final volume with ultrapure water. The concentrations of Pb and Cd were then quantified using a flame atomic absorption spectrophotometer (AA-680, Shimadzu, Kyoto, Japan).

### Physicochemical analysis of rhizosphere

The air-dried soil was sieved through 20- and 100-mesh nylon sieves. Soil samples that passed through the 20-mesh sieve were used for the determination of soil pH, soil organic matter (SOM), cation exchange capacity (CEC), urease activity (Ure), and catalase activity (S-CAT). Soil pH was measured using the potentiometric method, whereas SOM content was determined via potassium dichromate oxidation followed by titration. CEC was assessed using the barium chloride-sulfuric acid exchange method, and S-CAT activity was quantified using the potassium permanganate titration method. In addition, ammonium nitrogen (AN) and nitrate nitrogen (NN) concentrations were analyzed using a COMIN test kit.

### Sample digestion and metal analysis

A 0.5-g aliquot of air-dried soil, sieved through a 100-mesh sieve, was subjected to cold digestion overnight with 8 mL of aqua regia (HNO₃/HCl, v/v = 1:3). The following day, the sample underwent further digestion in a heating oven with the addition of HClO₄ until the formation of a grayish–white residue, accompanied by white fumes and a final solution volume of approximately 3 mL. After digestion, the sample was diluted with ultrapure water and analyzed for Pb and Cd concentrations using a flame atomic absorption spectrophotometer (AA-680, Shimadzu, Kyoto, Japan).

### Soil physicochemical and biological parameters

The soil organic carbon (SOC) content was determined using potassium dichromate oxidation. Total nitrogen (TN) content was estimated with a TOC analyzer (Multi N/C 3100 TOC, Analytik, Jena, Germany). Soil moisture (SM) was analyzed by weighing the soil and calculating the mass lost after oven drying at 105°C until the weight remained stable (24 h). The soil pH was determined with a soil-to-water ratio of 1:2.5 (w/v) using a pH meter (FE20, METTLER TOLEDO, China). Ca_2_C content was measured using an atomic absorption spectrophotometer (AA-680, Shimadzu, Kyoto, Japan).

### DNA extraction and polymerase chain reaction amplification

Total microbial genomic DNA was extracted from soil samples using the E. Z. N. A.^®^ soil DNA Kit (Omega Bio-tek, Norcross, GA, United States) according to the manufacturer’s instructions. The quality and concentration of DNA were determined by 1.0% agarose gel electrophoresis and a spectrophotometer (NanoDrop 2000,Thermo Scientific, United States) and kept at −80°C prior to further use. The hypervariable region V3–V4 of the bacterial 16S ribosomal RNA (rRNA) gene was amplified with primer pairs 338F (5′-ACTCCTACGGGAGGCAGCAG-3′) and 806R (5′-GGACTACHVGGGTWTCTAAT-3′) (Liu et al., 2016) by PCR thermocycler (T100, BIO-RAD, United States). The polymerase chain reaction (PCR) reaction mixture included 4-μL 5 × Fast Pfu buffer, 2-μL 2.5-mM dNTPs, 0.8-μL each primer (5 μM), 0.4-μL Fast Pfu polymerase, 10 ng of template DNA, and ddH_2_O to a final volume of 20 μL. PCR amplification cycling conditions were as follows: initial denaturation at 95°C for 3 min, followed by 27 cycles of denaturing at 95°C for 30 s, annealing at 55°C for 30 s and extension at 72°Cfor 45 s, and a single extension at 72°C for 10 min, and ended at 4°C. The PCR product was extracted from a 2% agarose gel and purified using the PCR Clean-Up Kit (YuHua, Shanghai, China) according to the manufacturer’s instructions and quantified using fluorometer (Qubit 4.0, Thermo Fisher Scientific, Qubit 4.0 (Thermo Fisher Scientific, United States)).

### Illumina sequencing

Purified amplicons were pooled in equimolar amounts and paired-end sequenced on an Illumina NextSeq 2000 platform (Illumina, San Diego, CA, United States) according to standard protocols by Majorbio Bio-Pharm Technology Co., Ltd. (Shanghai, China). The raw sequencing reads were deposited into the National Center for Biotechnology Information (NCBI) Sequence Read Archive (SRA) database (accession number: PRJNA1293673).

### Amplicon sequence processing and analysis

After demultiplexing, the resulting sequences were quality-filtered with fastp version 0.19.6 (Chen et al., 2018) software and merged with FLASH version 1.2.11 (Magoc and Salzberg, 2011). Then the high-quality sequences were de-noised using DADA2 (Edgar, 2013) plugin in the Qiime2 version 2020.2 (Wang et al., 2007) platform pipeline with recommended parameters, which obtains single nucleotide resolution based on error profiles within samples. DADA2 denoised sequences are usually called amplicon sequence variants (ASVs). To minimize the effects of sequencing depth on alpha-and beta-diversity measure, the number of sequences from each sample was rarefied to 20,000, which still yielded an average good’s coverage of 97.90%. Taxonomic assignment of ASVs was performed using the Naive Bayes consensus taxonomy classifier implemented in Qiime2 and the SILVA 16S rRNA database version 138. The metagenomic function was predicted using Phylogenetic Investigation of Communities by Reconstruction of Unobserved States (PICRUSt2) (Douglas et al., 2020) based on ASV representative sequences. PICRUSt2 is a software containing a series of tools as follows: HMMER—biosequence analysis using profile hidden Markov models—was used to align ASV representative sequences with reference sequences. The evolutionary Placement Algorithm ng (EPA-ng) and Gappa were used to place ASV representative sequences into a reference tree. The castor was used to normalize the 16S gene copies. MinPath was used to predict gene family profiles and locate them in the gene pathways. The entire analysis process was according to the protocols of PICRUSt2.

### Bioinformatic analysis

Bioinformatic analysis of the soil microbiota was carried out using the Majorbio Cloud platform.^1^ Based on the ASVs information, rarefaction curves and alpha-diversity indices, including observed ASVs, Chao1 richness, Shannon index, and Good’s coverage, were calculated with Mothur version 1.30.1 (Schloss et al., 2009). The similarity among the microbial communities in different samples was determined by principal coordinate analysis (PCoA) based on Bray–Curtis dissimilarity using the Vegan version 2.5–3 package. The permutational multivariate analysis of variance (PERMANOVA) test was used to assess the percentage of variation explained by the treatment, along with its statistical significance, using the Vegan version 2.5–3 package. The linear discriminant analysis (LDA) effect size (LEfSe) (Segata et al., 2011)^2^ was performed to identify the significantly abundant taxa (phylum to genera) of bacteria among the different groups (LDA score > 2, *p* < 0.05). Since there is a multicollinearity problem among the 12 soil physicochemical properties, the variance inflation factor (VIF) for each variable was estimated using the vif function in the car package.^3^ The distance-based redundancy analysis (db-RDA) was performed using the Vegan version 2.5–3 package to investigate the effect of soil physicochemical properties on soil bacterial community structure. Forward selection was based on Monte Carlo permutation tests (permutations = 9,999). Values of the *x-* and *y*-axes and the length of the corresponding arrows represented the importance of each soil physicochemical property in explaining the distribution of the taxon across communities. Linear regression analysis was applied to determine the association between major physicochemical properties identified by db-RDA analysis and microbial alpha-diversity indices. The co-occurrence networks were constructed to explore the internal community relationships across the samples (Barberan et al., 2012). A correlation between two nodes was considered to be statistically robust if the Spearman’s correlation coefficient was over 0.6 or less than −0.6, and the *p*-value was less than 0.01.

### Metabolome extraction and analysis

Metabolome extraction and analysis were performed by a commercial company (Metware Biotechnology Co., Ltd., Wuhan, China). Metabolite profiling was conducted through a non-targeted metabolomic approach by Wuhan Metware Biotechnology Co., Ltd. (Wuhan, China),^4^ with the sample extracts analyzed using an ultra-performance liquid chromatography coupled with electrospray ionization and tandem mass spectrometry (UPLC-ESI-MS/MS) system (UPLC, Shim-pack UFLC SHIMADZU CBM30A system^5^; MS, Applied Biosystems 4500 Q TRAP^6^).

In brief, the sample metabolome extraction, the freeze-dried rhizosphere soil of *S. matsudana* was ground into powder using a mixer mill (MM 400, Retsch) with zirconia beads for 1.5 min at 30 Hz. The tissue powder (0.1 g) was weighed and extracted overnight at 4°C with 0.6 mL 70% (v/v) aqueous methanol prior to centrifugation at 10,000 g for 10 min, followed by absorption (CNWBOND Carbon-GCB SPE Cartridge, 250 mg, 3 mL; ANPEL, Shanghai, China^7^) and filtration (SCAA-104, 0.22 μm pore size; ANPEL, Shanghai, China, see Footnote 7).

Data collection and verification were performed via the Analyst software version 1.6.3 (AB Sciex). The metabolites were identified using the Metware database (MWDB). The metabolite abundances were quantified according to their peak areas. The data were deposited into the NCBI database (accession number: MTBLS12749).

Metabolites were considered to have differentially accumulated when the variable importance in projection (VIP) score was ≥1 and the absolute log2(fold change) was ≥1.

### Statistical analysis

All experimental data in this study were obtained from four independent replicates, with results presented as mean ± standard deviation (SD). Biochemical and physiological data were subjected to one-way analysis of variance (ANOVA), with statistical significance set at *p* < 0.05. Statistical analyses were conducted using IBM Statistical Package for Social Sciences (SPSS) version 26.0.0 software, and intergroup differences were evaluated by Student’s *t*-test.^8^ Levels of statistical significance were defined as follows: *p < 0.05. Data visualization was generated using Origin 2021 software (OriginLab Corporation).

For multi-omics analysis, transcriptomic and metabolomic data processing was carried out on the Majorbio Cloud Platform.^9^ Cluster heatmap visualization was conducted using TBtools software (Chen et al., 2020). An integrative analysis of transcriptome–metabolome correlations was performed using Two-way Orthogonal Partial Least Squares (O2PLS) analysis, supplemented by Pearson correlation algorithms (Meng et al., 2022).

## Results

### Nitrogen fertilization enhances *Salix matsudana* growth and heavy metal absorption

**Figure 1:**
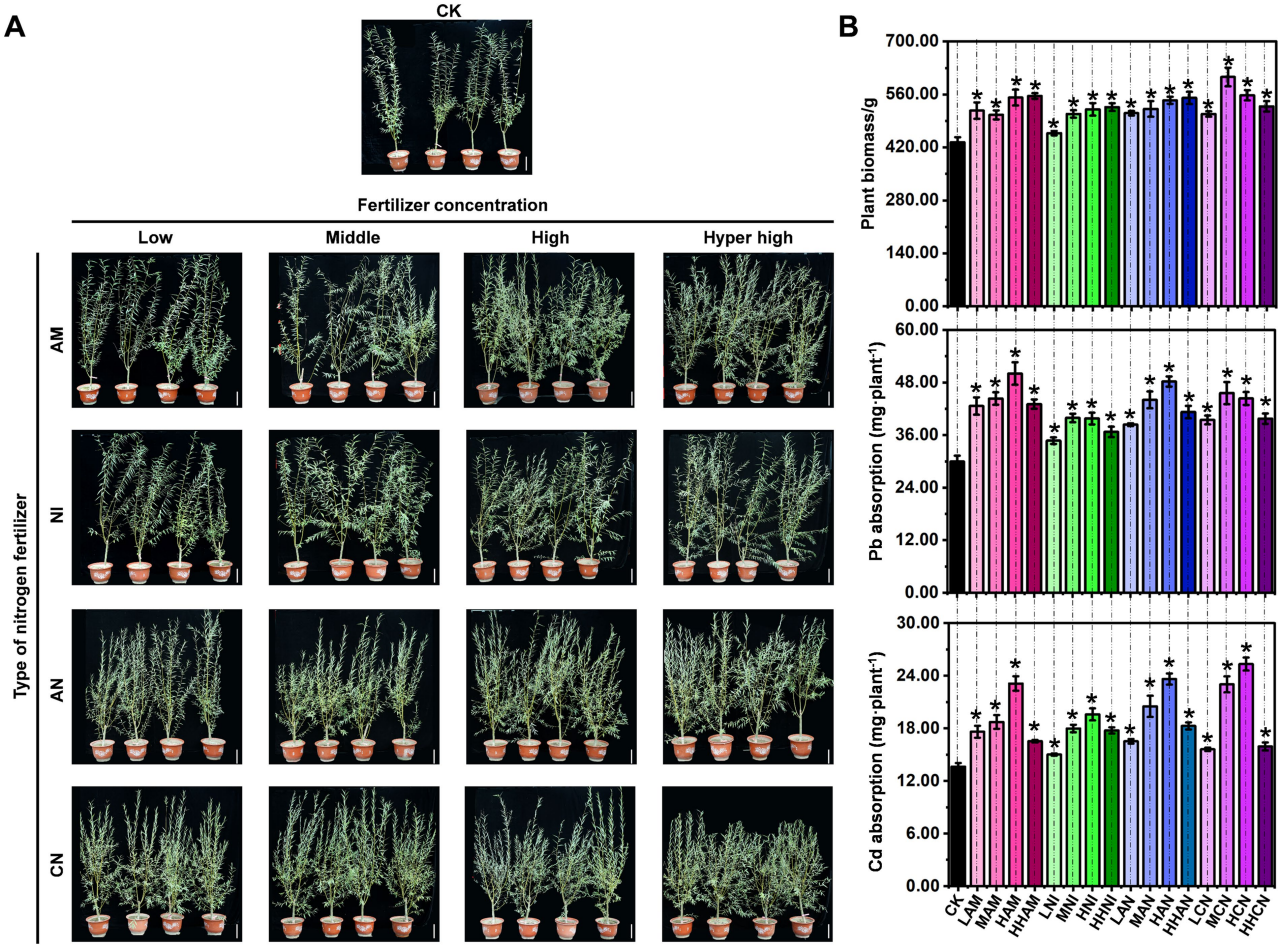
Effects of nitrogen fertilizer on *Salix matsudana* growth and uptake of heavy metals. **(A)** *S. matsudana* was transplanted into heavy metal-contaminated soil supplemented with four types of nitrogen fertilizers: ammonium nitrogen (AM), nitrate nitrogen (NI), a 1:1 mixture of ammonium and nitrate (AN), and urea nitrogen (CN). Each fertilizer was applied at four concentration levels (low, medium, high, and hyper-high). Scale bar: 10 cm. The control (CK) group consisted of *S. matsudana* grown in heavy metal-contaminated soil without nitrogen fertilization. **(B)** Plant biomass and Pb/Cd accumulation in *S. matsudana* under different nitrogen treatments. Data are presented as mean ± SD (*p < 0.05, Student’s *t*-test).

As a widely utilized landscaping species, *S. matsudana* exhibits variable growth rates when cultivated in nitrogen-fertilized soils. Although nitrogen fertilization is well recognized for its role in promoting plant growth, the specific impacts of different nitrogen fertilizer types on *S. matsudana* remain insufficiently understood. To address this knowledge gap, we designed a controlled experiment to investigate the growth response of *S. matsudana* to four types of nitrogen fertilizer: ammonium nitrogen (AM), nitrate nitrogen (NI), a 1:1 ammonium–nitrate mixture (AN), and urea nitrogen (CN). These fertilizers were applied at four concentration levels (low, medium, high, and hyper-high) in heavy metal-contaminated soil (Table 1). Initial observations indicated that *S. matsudana* exhibited enhanced growth with increasing fertilizer concentrations under potted conditions (Figure 1A). Subsequent biomass measurements confirmed this trend, revealing significant growth promotion across all nitrogen treatments (Figure 1B; Supplementary Text S1). Notably, optimal growth responses were observed under specific fertilizer regimes: high-level AM (551.80 g), hyper-high-level NI (556.15 g), hyper-high-level AN (550.85 g), and low-level CN treatments (508.31 g) demonstrated the most pronounced effects on plant development.

As plants grow, they absorb mineral nutrients from the soil. However, it remains unclear whether nitrogen fertilization enhances the uptake of heavy metals by *S. matsudana* during its growth. To investigate this relationship, we quantified Pb and Cd accumulation in *S. matsudana* (Figure 1A). The results indicated that Pb and Cd uptake by *S. matsudana* increased following the application of four different types of nitrogen fertilizer (Figure 1B). The highest levels of Pb and Cd accumulation were observed under high nitrogen fertilization, high-level AM (Pb: 50.04 mg·plant^-1^; Cd: 23.13 mg·plant^-1^), high-level NI (Pb: 39.77 mg·plant^-1^; Cd: 19.61 mg·plant^-1^), and high-level AN (Pb: 48.23 mg·plant^-1^; Cd: 23.64 mg·plant^-1^), high-level CN (Pb: 44.35 mg·plant^-1^; Cd: 25.35 mg·plant^-1^) (Supplementary Text S1). However, when nitrogen fertilizer was applied at excessively high concentrations, Pb and Cd uptake decreased (Figure 1B). These findings suggest that nitrogen fertilization not only promotes the *S. matsudana* growth but also enhances its capacity for Pb and Cd absorption. The most favorable nitrogen concentration for this enhancement was identified under high-level application.

### Nitrogen fertilization enhances heavy metal accumulation in *Salix matsudana* root systems

**Figure 2:**
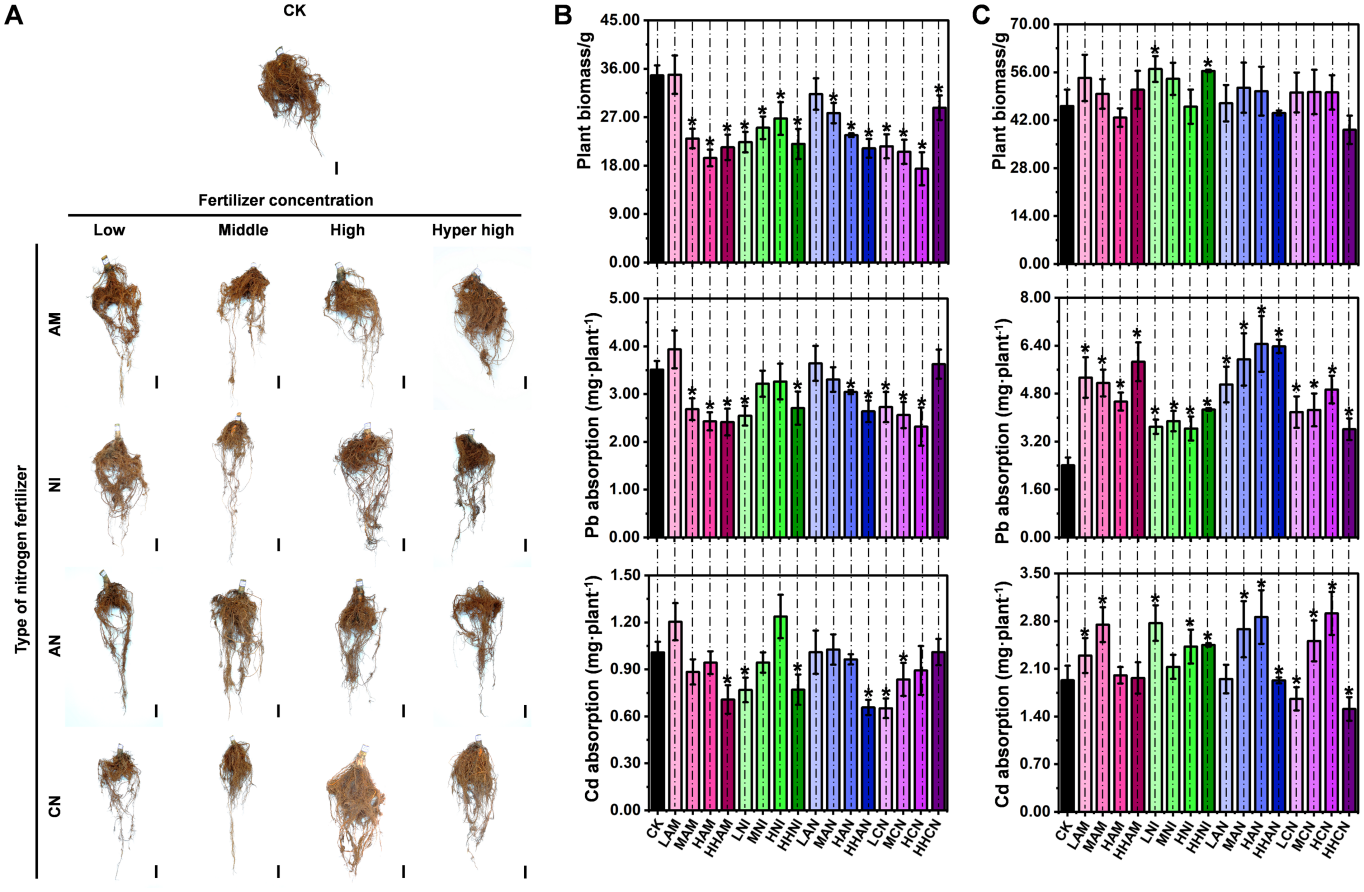
Nitrogen fertilizer enhances heavy metal accumulation in *Salix matsudana* roots. **(A)** Phenotypic observation of *S. matsudana* roots grown in heavy metal-contaminated soil with or without nitrogen fertilizer supplementation. Control (CK) represents roots grown in heavy metal-contaminated soil without nitrogen fertilizer application. Scale bar, 1 cm. **(B)** Biomass and heavy metal (Pb and Cd) accumulation in primary roots of *S. matsudana* grown in heavy metal-contaminated soil supplemented with four different nitrogen fertilizers. **(C)** Biomass and heavy metal (Pb and Cd) accumulation in lateral roots of *S. matsudana* under the same treatment conditions as discussed in subpart **(B)**. Data are presented as mean ± SD; *p < 0.05 (Student’s *t*-test).

Plants primarily absorb mineral nutrients from the soil through their root systems. As illustrated in Figure 1, nitrogen fertilization enhances both the *S. matsudana* growth and its uptake of heavy metals. To investigate the specific effects of nitrogen fertilization on root development, we isolated *S. matsudana* roots and conducted a systematic analysis of their growth patterns. The results showed that *S. matsudana* roots exhibited significantly enhanced growth vigor in soils amended with any of the four tested nitrogen fertilizers (Figure 2A). Moreover, increasing nitrogen concentrations were associated with more pronounced root proliferation. For a more detailed assessment of nitrogen’s impact on root architecture, we categorized the root system into primary and lateral roots for quantitative analysis. Biomass measurements revealed that primary root growth was inhibited in soils treated with ammonium nitrogen fertilizer (AM). Similarly, the biomass of primary roots in nitrate nitrogen (NI)-, ammonium–nitrate mixture (AN)-, and urea nitrogen (CN)-treated soils was consistently lower than that in the control group (Figure 2B; Supplementary Text S2). In contrast, lateral root biomass exhibited a differential response: treatments with low-level AM fertilizer, as well as low-and hyper-high-level NI fertilizer, resulted in significantly greater lateral root biomass compared to the control (Figure 2C).

To further investigate whether nitrogen fertilization enhances uptake of heavy metals in *S. matsudana* roots, we quantified Pb and Cd accumulation in both primary and lateral roots. In primary roots, significantly increased Pb accumulation was observed only under low-level AM treatment compared to the control. In contrast, elevated Cd accumulation occurred under both low-level AM and high-level NI treatments (Figure 2B). All other nitrogen fertilizer applications resulted in reduced Pb and Cd accumulation in primary roots relative to the control group. Regarding lateral roots, Pb accumulation was enhanced across all four nitrogen fertilizer treatments when compared with the control (Figure 2C; Supplementary Text S3). Similarly, Cd accumulation in lateral roots was significantly increased under multiple treatment conditions: low-and medium-level AM; low-, medium-, high-, and hyper-high-level NI; medium-and high-level AN; and medium-and high-level CN (Figure 2C). These findings indicate that nitrogen fertilization does not promote root growth in *S. matsudana*, but it can enhance Pb and Cd uptake at specific concentration levels.

### Nitrogen fertilizer improved the physicochemical properties of heavy metal-contaminated soil

**Figure 3:**
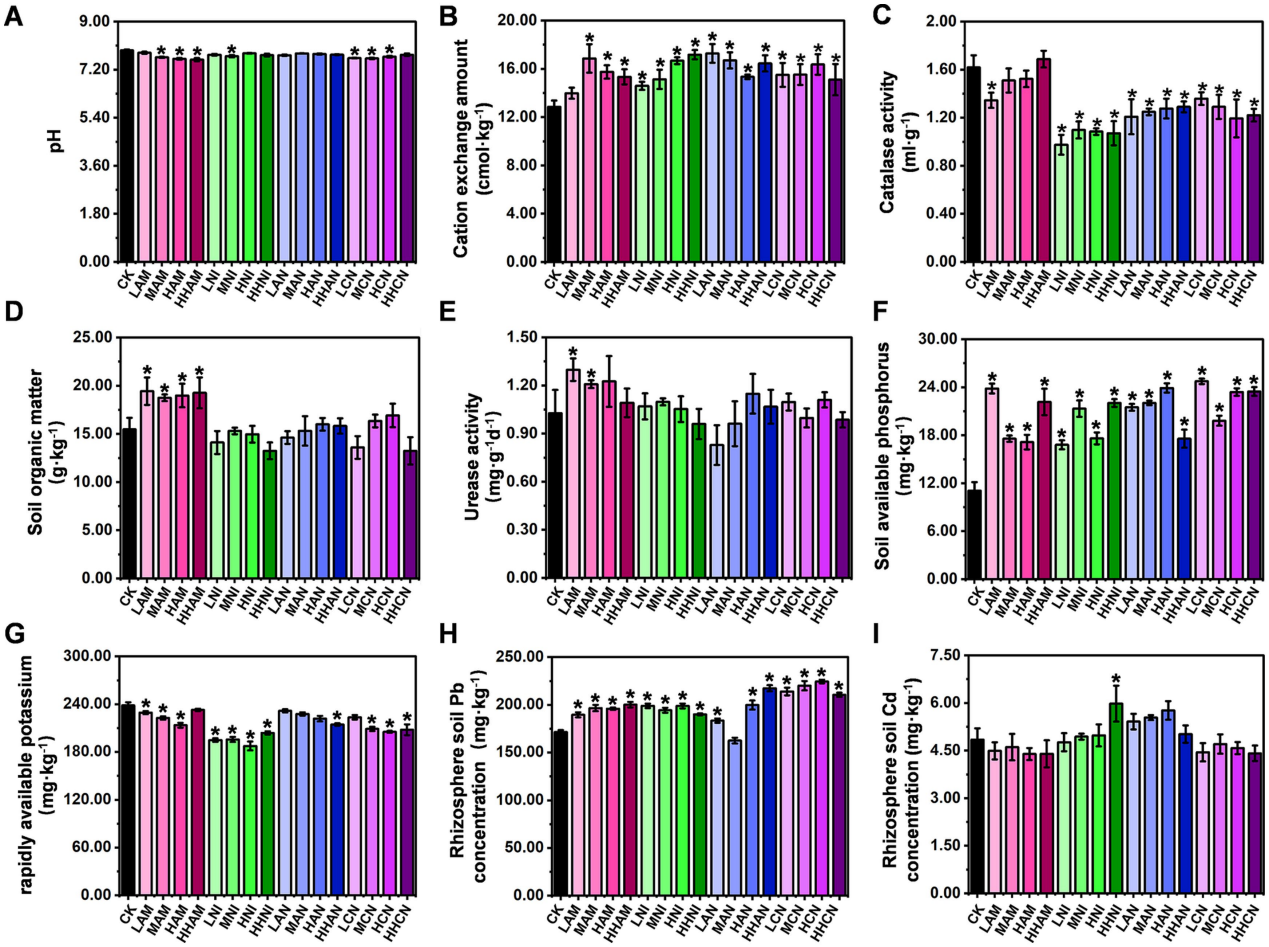
Effects of nitrogen fertilizer on the physicochemical properties of heavy metal-contaminated soil. **(A)** Soil pH after nitrogen fertilizer application. **(B)** Soil cation exchange capacity (CEC) following nitrogen fertilizer treatment. **(C)** Soil catalase activity in response to nitrogen fertilizer. **(D)** Soil organic matter content after nitrogen fertilizer amendment. **(E)** Urease activity in soil treated with nitrogen fertilizer. **(F)** Soil phosphorus content following nitrogen fertilizer application. **(G)** Soil potassium content after nitrogen fertilizer treatment. **(H)** Pb concentration in plant roots under nitrogen fertilization. **(I)** Cd concentration in plant roots under nitrogen fertilization. Data are presented as mean ± SD (*p < 0.05, Student’s *t*-test).

As illustrated in Figures 1, 2, nitrogen fertilization significantly enhances both the *S. matsudana* growth and its uptake of heavy metals. Given that plants absorb nutrients from the soil, this raises an important scientific question: How do the physicochemical properties of heavy metal-contaminated soil change when nitrogen fertilization stimulates nutrient absorption by plants? To address this question, we conducted an experiment using heavy metal-contaminated soil treated with four concentrations (low, medium, high, and hyper-high) of four nitrogen fertilizer types (AM, NI, AN, and CN), with *S. matsudana* planted as the test species. Subsequently, we analyzed the soil’s physicochemical properties based on the results shown in Figures 1, 2. The pH of heavy metal-contaminated soil decreased following the application of nitrogen fertilizers, specifically at medium, high, and hyper-high levels of AM (ammonium sulfate), medium levels of NI (nitrate), and low, medium, and high levels of CN (calcium nitrate) (Figure 3A; Supplementary Text S4). Additionally, all four nitrogen fertilizers—AM, NI, AN (ammonium nitrate), and CN—enhanced cation exchange capacity (CEC). Notably, the medium level of AM, hyper-high level of NI, low level of AN, and high level of CN exhibited the most pronounced effects on CEC improvement (Figure 3B). Catalase activity was significantly reduced under low-level AM treatment, as well as across all four levels of NI, AN, and CN application (Figure 3C). In contrast, organic matter content increased exclusively in response to AM treatment at all four levels, whereas NI, AN, and CN showed no significant effects (Figure 3D). Urease activity was enhanced only by low-and medium-level AM treatments, with no observable changes in other treatment groups (Figure 3E). Further analysis of soil nutrients revealed that all nitrogen fertilizers increased phosphorus content (Figure 3F). However, potassium content was negatively affected by low-, medium-, and high-level AM treatments, all levels of NI, hyper-high-level AN, and medium-, high-, and hyper-high-level CN treatments (Figure 3G). Regarding uptake of heavy metals, root Pb concentration increased under all nitrogen fertilizer treatments except for medium-level AN (Figure 3H). In contrast, root Cd concentration remained unchanged across all treatments except for hyper-high-level AN, which showed a significant increase (Figure 3I). Collectively, these findings demonstrate that nitrogen fertilizers can effectively modify the physicochemical properties of soils contaminated with heavy metals.

### Nitrogen fertilization enhanced rhizospheric microbia microbes

**Figure 4:**
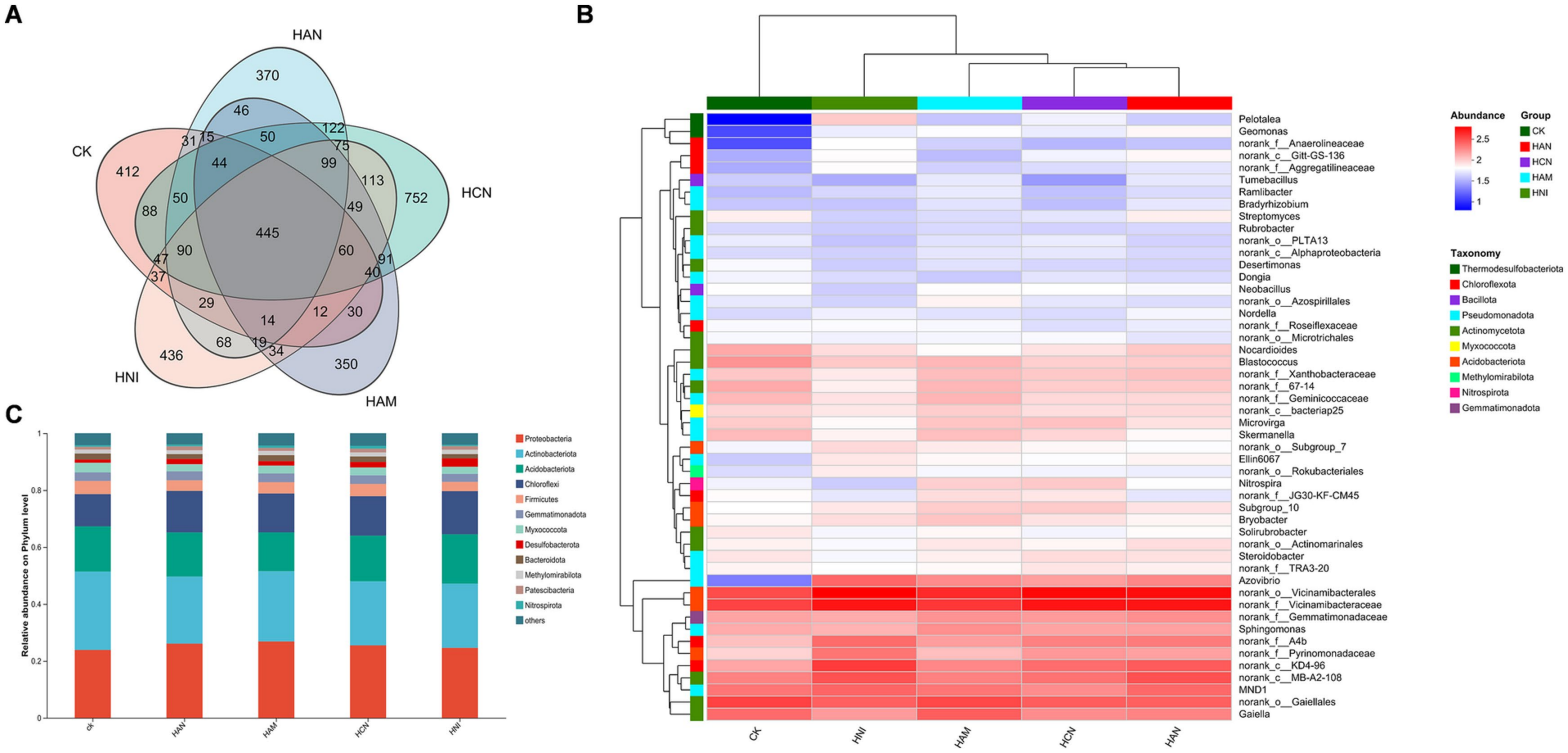
Effects of nitrogen fertilization on rhizospheric bacterial communities in *Salix matsudana* grown in heavy metal-contaminated soil. **(A)** Venn diagram illustrating the shared and unique bacterial taxa across (ASVs) four nitrogen fertilizer treatments. **(B)** Heatmap displaying the taxonomic composition of rhizosphere bacterial communities under different nitrogen fertilization regimes. **(C)** Relative abundance of dominant bacterial phyla in the rhizosphere of *S. matsudana* following nitrogen fertilizer application.

**Figure 5:**
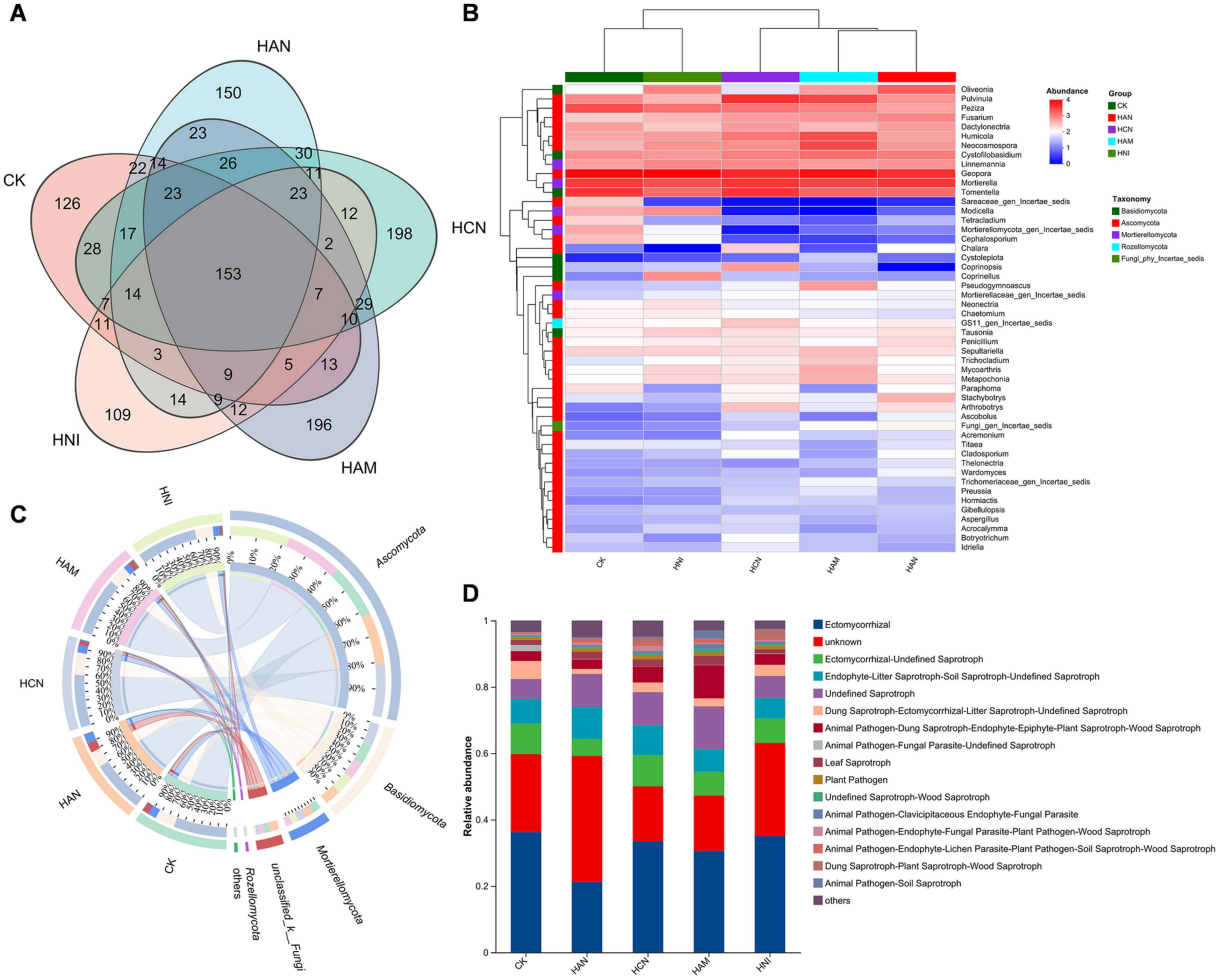
Influence of nitrogen fertilization on rhizosphere fungal communities in *Salix matsudana* grown in heavy metal-contaminated soil. **(A)** Venn diagram demonstrating the shared and unique fungal operational taxonomic units (ASVs) across four nitrogen fertilizer treatments. **(B)** Heatmap illustrating the composition of rhizosphere fungal communities under different nitrogen fertilization regimes. **(C)** Circos plot depicting the structure of fungal communities in nitrogen-amended soils. **(D)** Relative abundance of dominant fungal taxa in the rhizosphere of *S. matsudana* under nitrogen fertilization.

Nitrogen fertilization significantly enhances the *S. matsudana* growth and facilitates uptake of heavy metals through the root system, while simultaneously improving the physicochemical properties of heavy metal-contaminated soil (Figures 1–3). Given the abundance of microorganisms in the rhizosphere, we aimed to investigate whether nitrogen fertilization influences rhizospheric microbial communities in contaminated soils. To address this objective, we performed high-throughput sequencing of *S. matsudana* rhizosphere microbial communities (Supplementary Figure S2). Four types of nitrogen fertilizers were applied at high concentrations, along with an unfertilized control group, as high nitrogen levels were previously found to be more effective in promoting plant growth and heavy metal accumulation. Subsequent microbiome analysis revealed that nitrogen fertilization significantly altered both the composition and intergroup diversity of *S. matsudana* rhizosphere microorganisms (Supplementary Figure S3). Notably, different nitrogen fertilizers promoted distinct bacterial community structures (Figure 4A; Supplementary Text S5), with Proteobacteria, Actinobacteriota, Acidobacteriota, and Chloroflexi identified as the dominant phyla (Figures 4B,C). Similarly, nitrogen fertilization influenced fungal community structure (Figures 5A,B; Supplementary Text S5), with Ectomycorrhizal fungi representing the most abundant functional group (Figures 5C,D). Further analysis demonstrated a correlation between rhizosphere microbial composition and soil pH (Supplementary Figure S4), and Mantel tests confirmed a significant association between nitrogen fertilization and key soil physicochemical properties (Supplementary Figure S5). Additionally, phylogenetic evolutionary analysis provided insights into the role of nitrogen fertilization in shaping rhizosphere microbial dynamics (Supplementary Figure S6). Collectively, these findings demonstrate that nitrogen fertilization not only enhances plant growth and heavy metal remediation but also modulates the structure and functional dynamics of rhizosphere microbial communities.

### Metabolomic analysis of rhizosphere soil under different nitrogen fertilization regimes

**Figure 6:**
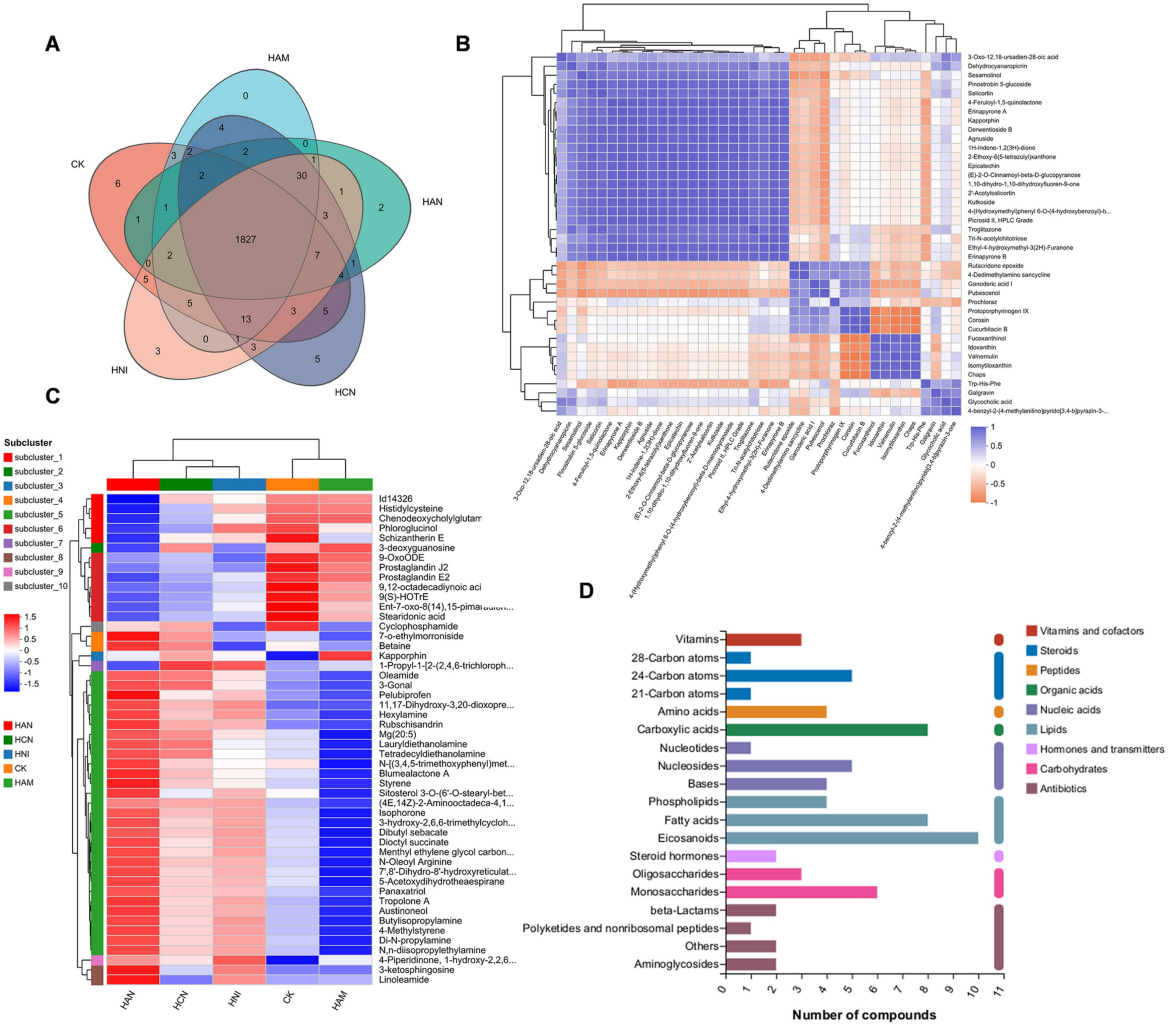
Metabolomic analysis of the effects of nitrogen fertilization on the rhizosphere soil of *Salix matsudana* grown in heavy metal-contaminated environments. **(A)** Venn diagram depicting the number of differentially accumulated metabolites (DAMs) in the rhizosphere soil of *S. matsudana* across four nitrogen fertilizer treatments. **(B)** Correlation analysis of differentially accumulated metabolites in the rhizosphere soil of *S. matsudana*. **(C)** Heatmap illustrating the relative abundance of differentially accumulated metabolites in the rhizosphere soil of *S. matsudana* under four nitrogen fertilizer regimes. **(D)** KEGG pathway enrichment analysis of differentially accumulated metabolites in the rhizosphere soil of *S. matsudana* under varying nitrogen fertilizer applications.

Nitrogen fertilizer application significantly influenced the rhizosphere metabolome of *S. matsudana* (Figure 1). To characterize the metabolic profiles of the rhizosphere under different nitrogen fertilization regimes, we collected soil samples from various treatment groups and conducted metabolomic profiling. First, we assessed the quality of the sequencing data. A global evaluation of the metabolomic experiment, including Pearson’s correlation coefficients and principal component analysis (PCA), confirmed that the samples exhibited minimal technical variation and were suitable for downstream analysis (Supplementary Figure S7). Next, we analyzed the metabolic profiles of the rhizosphere under different nitrogen fertilizer treatments. Metabolomic profiling revealed distinct patterns across treatment groups (Figure 6A). In the control group, 1948 metabolites were detected, while the nitrogen-treated groups showed varying numbers: 1884 in HAN, 1912 in HCN, 1893 in HAM, and 1904 in HNI. Notably, only a small subset of metabolites was unique to each group: 6 (0.31%) in the control, 2 (0.10%) in HAN, 5 (0.26%) in HCN, 3 (0.15%) in HNI, while the HAM group exhibits no unique metabolites. In contrast, 1,827 metabolites (94.03%) were shared across all groups (Supplementary Text S6). Subsequently, we further examined the relationships among differentially accumulated metabolites (DAMs). As illustrated in Figure 6B, some DAMs exhibited positive correlations, while others showed negative correlations. Hierarchical clustering of these metabolites revealed distinct patterns, with the DAMs segregating into 10 clusters based on their abundance profiles (Figure 6C; Supplementary Figure S8).

To further explore the metabolomic profile of *S. matsudana* rhizosphere soil under varying nitrogen fertilization regimes, we performed a Kyoto Encyclopedia of Genes and Genomes (KEGG) pathway enrichment analysis of the differentially accumulated metabolites. As shown in Figure 6D, these metabolites were predominantly linked to several biochemical categories, such as vitamins, steroids, organic acids, nucleic acids, lipids, hormones, carbohydrates, and antibiotics. Additionally, we constructed a metabolic pathway network (Supplementary Figure S9) to illustrate the key biological pathways associated with these metabolites. The results of these pathway analyses indicate notable correlations with the metabolic characteristics of *S. matsudana* rhizosphere soil. Our findings demonstrate that the four nitrogen fertilizer treatments have distinct influences on the soil metabolome of *S. matsudana*.

## Discussion

*S. matsudana* has emerged as a promising woody energy crop, particularly valued in landscaping due to its exceptional tolerance to heavy metals. This species possesses a unique ability to mitigate metal toxicity through various physiological and biochemical mechanisms (Zhang et al., 2025; Cao et al., 2018; Zou et al., 2022). For example, calcium (Ca) has been shown to alleviate Cd-induced toxicity in *S. matsudana* by regulating Cd mobility and enhancing detoxification processes within plant tissues (Zou et al., 2023). The genetic basis of *S. matsudana’*s heavy metal tolerance and growth characteristics has been extensively investigated, revealing its potential for coastal afforestation and the remediation of heavy metal-contaminated soils. Notably, its high biomass yield and strong metal tolerance render it an ideal candidate for phytoremediation, enabling the efficient uptake and stabilization of heavy metals in polluted environments. Further studies on the anatomical and physiological adaptations of *S. matsudana* under heavy metal stress have provided valuable insights. Variations in root anatomy and elemental distribution, for instance, play a crucial role in modulating metal uptake and tolerance, highlighting the species’ suitability for environmental remediation strategies. Given these attributes—rapid growth, significant biomass accumulation, and superior metal tolerance—*S. matsudana* is recognized as a dual-purpose species for sustainable bioenergy production and ecological restoration. In this study, we assessed its Pb and Cd absorption capacities (Figures 1, 2), further confirming its high tolerance to multiple heavy metals and supporting its application in phytoremediation.

The essential role of nitrogen fertilizers in promoting plant growth has been widely recognized, with numerous studies emphasizing the synergistic advantages of integrating chemical and biological strategies to enhance nitrogen availability and uptake efficiency. Research indicates that controlled-release fertilizers can markedly improve nitrogen use efficiency (NUE) by reducing leaching losses while maintaining a prolonged nutrient supply for plant development (Mikkelsen et al., 1994). These approaches are crucial for advancing sustainable agricultural systems. Further expanding this framework, investigations into plant growth-promoting rhizobacteria (PGPR) have demonstrated their capacity to reduce dependence on synthetic fertilizers without sacrificing crop productivity. For example, the combined applications of PGPR and reduced fertilizer rates in tomato cultivation resulted in growth and yield outcomes comparable to those achieved under conventional full-dose fertilization (Adesemoye et al., 2009), highlighting the potential of microbial inoculants as partial replacements for synthetic nitrogen inputs. Mechanistic studies on PGPR-mediated nitrogen uptake, employing isotopic tracing techniques such as 15 N-depleted ammonium sulfate, have confirmed that specific bacterial strains enhance the assimilation of fertilizer-derived nitrogen in plants (Adesemoye et al., 2010). These findings are consistent with observations in *Brassica rapa chinensis*, where inoculation with *Rhodopseudomonas palustris* supported normal growth under suboptimal nitrogen conditions, underscoring the significance of bioaugmentation in nutrient management (Wong et al., 2014). Crop-specific investigations, such as those involving *Bacillus pumilus* inoculation in tomatoes, have further clarified how plant growth-promoting bacteria (PGPB) optimize nitrogen utilization across varying fertilization regimes (Masood et al., 2020). In addition to nitrogen fixation, microbial contributions to phytohormone synthesis (e.g., indole-3-acetic acid, IAA) and root system development illustrate their diverse functional roles. Moreover, screenings of native bacterial isolates have highlighted microbial diversity as a key factor influencing enhanced shoot and root growth. Supporting this perspective, PGPR applications in wheat systems have been shown to activate soil nutrient pools, enabling vigorous plant growth even under reduced fertilizer regimes (Wang et al., 2020). Collectively, these findings advocate for an integrated strategy that combines chemical fertilization with microbial inoculation to improve NUE, reduce environmental impacts, and sustain agricultural productivity. Consistent with this body of evidence, our study reveals that nitrogen fertilization significantly enhances the growth performance of *S. matsudana* (Figure 1A), reinforcing the central importance of nitrogen in supporting plant development.

The influence of nitrogen fertilizers on soil improvement has become a focal point in agricultural research, with increasing attention directed toward their diverse impacts on soil health, microbial dynamics, and overall ecosystem functioning. This review synthesizes key findings from studies examining the complex interactions between nitrogen fertilization and soil properties, emphasizing both the advantages and challenges associated with sustainable agricultural practices. Recent investigations present contrasting viewpoints regarding the long-term consequences of nitrogen application. While its role in enhancing crop productivity is well established, multiple studies caution against potential negative outcomes, such as soil degradation and disturbances to microbial communities, highlighting the importance of balanced fertilizer management to maintain soil ecosystem integrity (Bijay-Singh, 2018). On the other hand, certain studies reveal context-specific benefits—for example, nitrogen application has been shown to promote microbial activity during phytoremediation, particularly in nickel phytoextraction by *Alyssum murale*, suggesting that strategic nitrogen use can enhance the effectiveness of remediation strategies for heavy metal-contaminated soils (Kanso et al., 2018). The relationship between nitrogen fertilization and soil microbial health is further clarified through studies on short-term fallow systems. Research indicates that nitrogen application may increase microbial biomass nitrogen, although it could also contribute to soil compaction, illustrating the complex trade-offs involved (Li et al., 2018). Similarly, synergistic effects have been observed when nitrogen fertilizers are applied in conjunction with organic amendments such as mulch, which improve plant growth conditions and soil structure (Wang et al., 2019). These integrated approaches underscore the value of combining inorganic and organic soil management techniques. The optimization of nitrogen application timing and dosage is also crucial for sustainable agriculture. It has been reported that reducing nitrogen input during early rice growth stages enhances nitrogen use efficiency (NUE) and improves soil nitrogen dynamics, offering a viable strategy to reduce environmental impacts while sustaining productivity. Furthermore, it has identified shifts in soil microbial communities linked to nitrogen fertilization, which subsequently affect crop quality. This is demonstrated in their study on tobacco, where specific microbial phylotypes responded positively to nitrogen inputs. Emerging strategies aimed at mitigating the adverse effects of nitrogen are gaining increasing attention. For instance, co-application of biochar has been found to alleviate nitrogen toxicity while simultaneously improving soil fertility and NUE (Khan et al., 2021). Similarly, nitrogen inhibitors have emerged as effective tools for enhancing soil multifunctionality by addressing microbial nitrogen limitations and improving soil quality indices (Yang et al., 2023). These developments illustrate the potential of complementary interventions to maximize the agronomic benefits of nitrogen. In our study, nitrogen fertilization was found to modify the physicochemical characteristics of heavy metal-contaminated soils, stimulate rhizosphere microbial activity, and alter soil metabolic pathways (Figures 4–6; Supplementary Figure S10). These results are consistent with existing literature, reinforcing the notion that the effects of nitrogen fertilization are highly dependent on environmental and management contexts.

**Figure 7:**
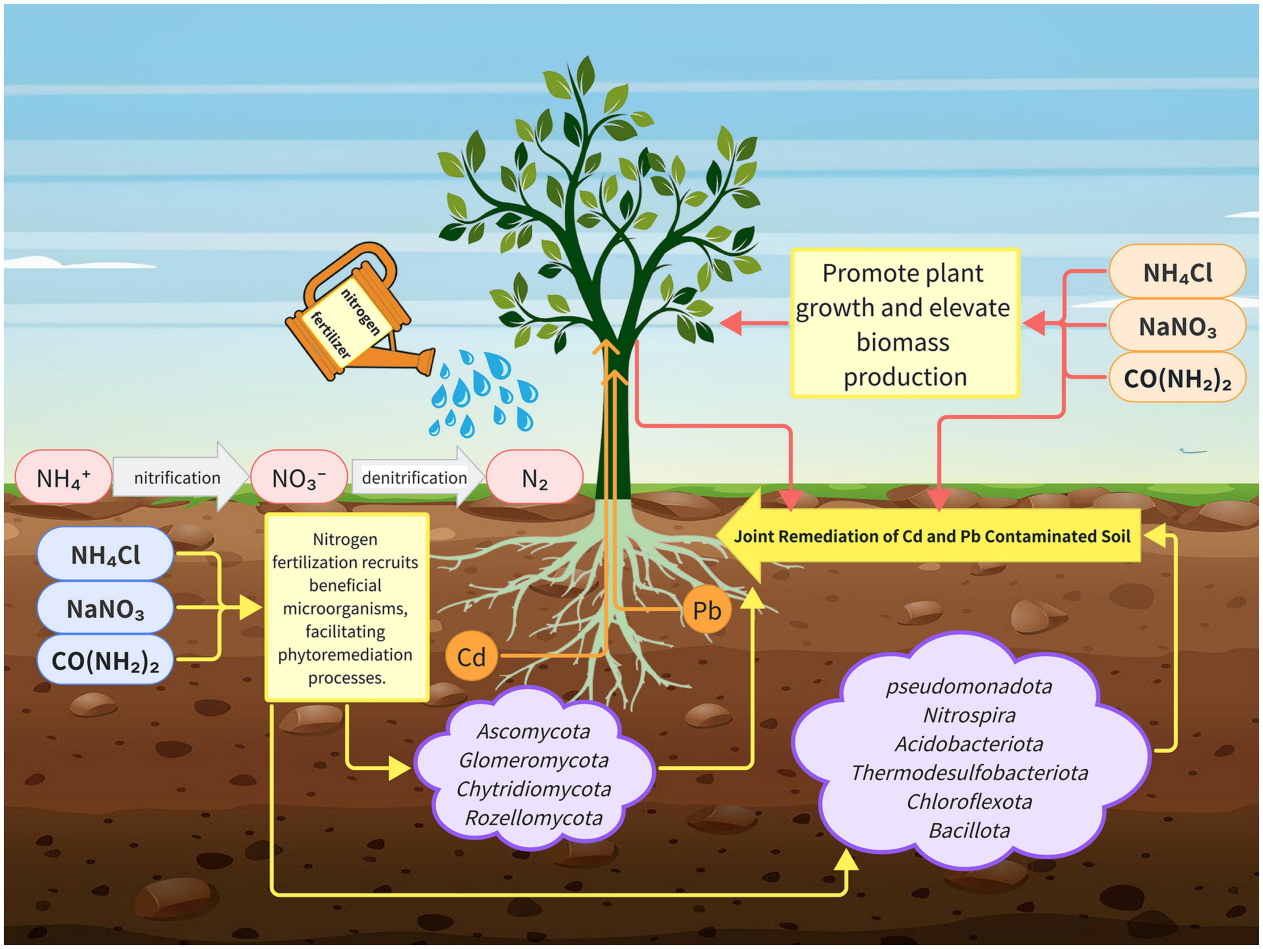
The proposed mechanism model of nitrogen fertilizer improves *Salix matsudana* growth and soil properties.

In summary, this study integrated a multi-omics approach with physiological analyses to elucidate the mechanisms through which nitrogen fertilization enhances the *S. matsudana* growth and its capacity for soil remediation. We assessed the growth performance of *S. matsudana* and the physiological characteristics of rhizosphere soil under varying nitrogen fertilizer levels, while also performing rhizosphere microbial community profiling and soil metabolomic analyses. Our results showed that high nitrogen fertilization significantly promotes plant growth and enhances the uptake of Pb and Cd. Specifically, root physiological assays indicated that nitrogen application increased Pb and Cd absorption compared to the control treatment. Moreover, analysis of rhizosphere microbial communities revealed that nitrogen fertilization enhances microbial activity, which in turn facilitates the uptake of heavy metals. Metabolomic profiling further supported these findings by demonstrating improved soil quality under high nitrogen conditions, consistent with the observed physiological improvements (Figure 7). These results provide new insights into the role of nitrogen fertilization in promoting both the *S. matsudana* growth and the soil restoration, offering a promising strategy for phytoremediation in heavy metal-contaminated environments.

## Data Availability

The datasets presented in this study can be found in online repositories. The names of the repository/repositories and accession number(s) can be found below: PRJNA1293673.

## Footnotes

^1^ https://cloud.majorbio.com

^2^ http://huttenhower.sph.harvard.edu/LEfSe

^3^ https://cran.r-project.org/web/packages/car/car.pdf

^4^ http://www.metware.cn/

^5^ http://www.shimadzu.com.cn/

^6^ http://www.appliedbiosystems.com/

^7^ http://www.anpel.com.cn/

^8^ http://www.physics.csbsju.edu/stats/

^9^ http://www.majorbio.com
